# Impact of human capital and social capital on employability of Chinese college students under COVID-19 epidemic—Joint moderating effects of perception reduction of employment opportunities and future career clarity

**DOI:** 10.3389/fpsyg.2022.1046952

**Published:** 2022-12-20

**Authors:** Yang Shiyuan, Yang Jinxiu, Xu Jingfei, Zhao Yuling, Yue Longhua, Li Houjian, Li Wei, Cheng Hao, He Guorong, Chen Juan

**Affiliations:** School of Humanities, Sichuan Agricultural University, Ya’an, China

**Keywords:** human capital, social capital, employability, perception reduction of employment opportunities, future career clarity, COVID-19 epidemic

## Abstract

This research constructed a relationship model between human capital, social capital, and the employability of college students. With two moderating variables introduced, the perception reduction of employment opportunities under the COVID-19 epidemic and future career clarity, this research studied the direct impact of human capital and social capital on the employability of college students and boundary conditions. Research data from 810 employed Chinese college graduates shows that both human capital and social capital have a positive and significant impact on the employability; the perception reduction of employment opportunities under the COVID-19 epidemic negatively regulates the relationship between human capital and the employability of college students; the future career clarity positively regulates the relationship between human capital and the employability of college students; the perception reduction of employment opportunities under COVID-19 epidemic and the future career clarity jointly regulate the relationship between human capital, social capital and the employability of college students. These conclusions enrich the relevant theoretical and practical research on the employability of college students under the COVID-19 epidemic.

## Introduction

Employment is the biggest people’s livelihood project, the people’s popular project, and the foundation project, and we must pay close attention to it. As one of the key groups for employment, college graduates have always been the focus of social attention. At the beginning of 2020, the COVID-19 epidemic swept the world, severely affecting the normal operation of my country’s economy and society, and the external employment environment is not optimistic. International Labour Organization data show that in 2021, global working hours will be reduced by 4.3% compared to pre-epidemic levels, which is equivalent to a reduction of 125 million full-time jobs (Chen, 2021). In addition, the scale of college graduates in my country is increasing year by year. In 2022, the number of college graduates will exceed 10.76 million, a year-on-year increase of 1.67 million, and both scale and increment will hit a record high (Zhou, 2022). The employment of college students has become the top priority in the employment field under the epidemic. Improving the employability of college students is the fundamental measure to solve the employment problem. Employability is a key node connecting colleges and the labor market, and it is directly related to whether the employment of college students can be realized and the quality of employment. Therefore, it poses a serious challenge to the cultivation of the employability of college graduates under the epidemic.

However, domestic scholars mostly focus on theoretical analysis of the concept, structure, and training strategies of college students’ employability, and lack the support of field research and relevant data on college students’ employability. Research methods are also not standardized enough. At present, the most representative definition of employability is Hillage and Pollard (1998). While foreign research objects on “employability” are mostly concentrated on industrial workers or general laborers, domestic research is mainly aimed at the group of college students (Guo, 2017). The employability of college students is a concept with rich connotations and continuous development (Wang and Sun, 2018). The lack of existing research will hinder a comprehensive understanding of the employability of college students with local characteristics. Employers usually measure the job seeker’s education level, work ability, work experience, and other human capital to determine whether the job seeker is a suitable employee, while social capital enables job seekers to obtain resources through social relations to achieve employment goals. Therefore, “personal ability” and “social capital” enable college students to actively respond to changes in the external working environment and tasks to be recognized by the labor market. Therefore, human capital and social capital have a positive impact on employability.

Furthermore, it is unscientific to separate the positive effects of human and social capital on employability from the environment and individuals. The outbreak of the COVID-19 epidemic has led to a decrease in employment opportunities and an increase in employment pressure. The Stress Cognitive Evaluation Theory holds that after evaluating the stress, if the stress is considered damaging and threatening, then the individual will tend to adopt a negative coping style (Folkman, 1986). As an external environmental factor, the perceived reduction of employment opportunities under COVID-19 will have an impact on employability (Mcquaid and Lindsay, 2005). When college students perceive a higher perception of reduction of employment opportunities, greater employment pressure, a pessimistic attitude toward employment, and a negative coping approach, the impact of human capital and social capital on improving employability will be smaller. In the process of job hunting for college students, future career clarity, as one of their characteristics, will also affect the relationship between human capital and social capital, and employability. In the current situation, a clear and easy-to-imagine future work can better provide targeted guidance and behavioral motivation for future-oriented behaviors. Job-seeking college students with a clear career development path will be more motivated and have more time to acquire the target occupation than those with a vague career development path, thereby strengthening the impact of their human capital and social capital on their employability (Locke and Latham, 2002; Locke, 2015; Ye et al., 2017). Therefore, this study constructs a model of the relationship between human capital, social capital, and college students’ employability, and attempts to demonstrate the situational moderating effects of perception reduction of employment opportunities under the COVID-19 epidemic and future career clarity in the relationship between human capital, social capital, and college students’ employability.

## Theoretical basis and research hypotheses

### The relationship between human capital and employability of college students

The capital that people spend on health care, education, training, and job migration is human capital (Schultz, 1990), the ability that is embodied in workers and provides them with permanent income. In a certain period, human capital is mainly manifested in the knowledge, skills, labor proficiency, and health status of workers (Zeng, 2019). Demographic variables, such as school rankings, majors, education levels, etc., can reflect human capital (Fugate et al., 2004). Zhang (2007) included grade rankings, scholarships, English ability, vocational qualification certificates, and work experience into the category of human capital. Employability refers to personal characteristics and attributes such as knowledge, skills, attitudes, and abilities that individuals need for initial employment, maintaining employment, and obtaining re-employment opportunities when necessary (Hillage and Pollard, 1998; Harvey, 2001; Berntson and Marklund, 2007; Hu, 2012; Zhang and Nie, 2015).

Human Capital Theory points out that human ability has an important impact on economic and social development, and research shows that college students’ human capital has a significant positive relationship with employment. Based on a large-scale sample survey on the employment status of college graduates across the country, Yue et al. (2004) showed that academic qualifications and professional performance are the most critical factors in determining the employment competitiveness of college graduates. Wang (2020) investigated the influence of two sub-dimensions of college students’ human capital, “educational learning status” and “participation in social practice” on their employment quality, and found that both educational learning status and participation in social practice would have a significant positive impact on the employment quality of college students. Employability is the guarantee of employment quality and competitiveness in the current situation, and college students’ human capital has a significant positive impact on employability (Hu and Shen, 2020). Human capital is a resource that can help individuals obtain other resources, and is an energy resource; while employability can create conditions for individuals to obtain key resources, and it is a conditional resource (Hobfoll and Lerman, 1989). According to the Resource Conservation Theory, when individuals have more resources, they are more able to acquire other resources. Therefore, the higher the stock of human capital that college students have through accumulation, the easier it is to change their disadvantaged position, and the more able they are to obtain good jobs. In summary:

*H1:* Human capital of college students has a positive impact on employability.

### The relationship between social capital and employability of college students

Bourdieu (1980) was the first to propose the concept of social capital, arguing that social capital is a collection of actual or potential resources that are related to a persistent network of mutually tacit or recognized relationships. Coleman (1988) defined it in terms of the function of social capital, arguing that social capital is a complex that constitutes certain aspects of the social structure and promotes the social behavior of individuals and others, and can contribute to the achievement of people’s goals.

Existing studies have shown that college students’ social capital has a positive impact on employment quality. Chen and Tan (2004) surveyed the employment situation of graduates in 14 colleges and universities and found that 92.8% of college graduates obtained employment information through their social capital, and as high as 90.1% of graduates believed that social relations had a great impact on employment. Wang (2020) examines the impact of college students’ social capital on their employment quality and finds that both dimensions of prior social capital and subsequent social capital have a significant positive impact on the employment quality of college students. The help of social capital to the employment of college students mainly comes from three aspects. The first is to help collect and screen employment information. Social capital can help employees to establish an extensive and reliable information network. Secondly, under the influence of social capital, the cost of interpersonal transactions is greatly reduced, the trust between the two parties is relatively high, and the chance of successful employment is greatly increased (Xu, 2002). Finally, social capital can also exert practical influence on applicants for job seekers, that is, “going through the back door,” which has become an unspoken professional rule in China’s relational society. Resource Conservation Theory states that people are always actively trying to build and maintain what they see as valuable resources. Employability is the ability that makes it easier for graduates to achieve career success and falls under the category of resources. The higher the level of social capital owned by an individual, the higher the level of occupational support it provides, the greater the help for employment, and the easier it is to gain a competitive advantage in the labor market. Therefore proposed:

*H2:* Social capital of college students has a positive impact on employability.

### Contingency effect of perception reduction of employment opportunities under the COVID-19 epidemic

Employment opportunities refer to the possibility of individuals obtaining job opportunities (Wang, 2016). Perception is an individual’s subjective feeling in the process of situational experience, and the individual’s subjective evaluation and judgment of things (Su et al., 2019). The perception reduction of employment opportunities under the COVID-19 epidemic refers to the feelings and judgments of job seekers about the degree of reduction in the target job opportunities under the background of the COVID-19 epidemic and a not optimistic employment environment. Different individuals have different feelings and judgments on whether they can get the target job and the quality of the job obtained through the evaluation of their employability and the experience of the employment environment.

Zhong and Wang (2020) believe that due to the dual impact of the economic downturn and the epidemic, companies have reduced human capital, many companies have reduced or canceled recruitment plans, and labor market demand has decreased. The epidemic has also delayed or canceled campus recruitment, the main employment channel for college graduates, resulting in some graduates becoming slack in their employment psychology. Coupled with the long waiting time at home, the possibility of panic and anxiety is also high. However, individuals differ in their perception reduction of employment opportunities in the labor market, depending on their knowledge and skills, work experience, and social connections. Some individuals believe that the epidemic has a great impact on employment opportunities, and some college students believe that the epidemic has less impact on the acquisition of employment opportunities. Su (2016) believes that the interaction between individuals and the environment, including identifying employment opportunities and obtaining employment opportunities, will have an impact on employability. The Stress Cognitive Evaluation Theory holds that if college students have a pessimistic attitude toward employment when they evaluate the current employment pressure as damage or threat, then college students will tend to respond negatively. Therefore, the perceived reduction of employment opportunities perceived by individuals will affect the relationship between human capital and social capital on employability, and an unfavorable external employment environment will weaken the positive impact of individual human capital and social capital on employability. When an individual perceives that the greater the perception reduction of employment opportunities, the fewer jobs suitable for him due to the epidemic, or the fewer opportunities for him to go for an interview, the human capital and social capital possessed by the individual at this time will be less helpful to employability. Therefore, the following assumptions are made:

*H3A:* The perception reduction of employment opportunities under the COVID-19 epidemic negatively adjusts the relationship between human capital and the employability of college students. The lower perception reduction of employment opportunities under the epidemic, the weaker the impact of human capital on employability.

*H3B:* The perception reduction of employment opportunities under the COVID-19 epidemic negatively adjusts the relationship between social capital and the employability of college students. The lower perception reduction of employment opportunities under the epidemic, the weaker the impact of social capital on employability.

### The contingency effect of college students’ future career clarity

Karoline (2012) proposed that future career clarity refers to the individual’s clarity and ease of imagining future jobs. It means that individuals have a clear understanding of their career goals, the way to achieve them, the methods needed to promote career development, the obstacles they may encounter, and the ways to deal with them (Guo et al., 2005). Occupational clarity focuses on one’s inner understanding of the occupation and whether the individual has a clear career-related series of questions, which reflects whether the job seeker has a clear job search goal and a clear idea of what job he wants to do (Lin et al., 2020).

The Goal Setting Theory points out that the individual’s action mode is determined by the individual’s psychological subconscious goals and objectives. A specific and clear goal can stimulate the individual’s motivation more than a vague goal, and a clear goal will prompt the individual to act in the direction pointed by (Leondari et al., 1998). Differences in how individuals compare their current self with their future self underlie active behavior (Gao et al., 2018). When individuals have a high sense of future career clarity, they will compare their current career situation with future expectations, thereby generating motivation to change or achieve self-development. And job seekers who lack clear job-seeking goals may spend more time trying out different ideas and thinking about future career development issues, which may reduce job-seeking behavior. Therefore, future career clarity will play a role in promoting the positive effects of human capital, social capital, and employability of college students. When college students’ future career clarity is higher, individuals may have a stronger job-seeking willingness and work toward their goals, thus showing more job-seeking behaviors. Therefore, the following assumptions are made:

*H4A:* Future career clarity of college students positively regulates the relationship between their human capital and employability. The stronger the college student's future career clarity, the greater the impact of human capital on employability.

*H4B:* Future career clarity of college students positively regulates the relationship between their social capital and employability. The stronger the college student's future career clarity, the greater the impact of social capital on employability.

### The joint effect of perception reduction of employment opportunities under the COVID-19 epidemic and college students’ future career clarity

The perception reduction of employment opportunities under the epidemic, future career clarity, and human capital and social capital may have a combined effect on employability. If and only if when there is a reasonable combination of perception reduction of employment opportunities under the epidemic, future career clarity, and human capital and social capital, it is possible to have high employability. Mcquaid and Lindsay (2005) believe that personal internal factors and external environmental factors are the main factors affecting employability, and it can be seen that the improvement of employability is the result of a combination of factors. When employment opportunities are reduced and the external employment environment is negative during the epidemic, the acquisition of employability may be restricted, thus preventing the positive impact of human capital and social capital on employability. In addition, future career clarity as an internal factor of the individual shows that the individual does not have a clear understanding and clear outlook. If the individual does not have a clear understanding of his future career goals, the individual will not have the direction and power of action, nor will he be able to reflect on and adjust his employment behavior, and his ability and external support will be powerless to obtain employability. When an individual perceives a lower perception reduction of employment opportunities under the epidemic, and when an individual has a higher future career clarity, the positive role of human capital and social capital in promoting employability can be fully utilized. Therefore, the following assumptions are made:

*H5A:* The lower perception reduction of employment opportunities under the COVID-19 epidemic and the stronger the future career clarity of college students, the stronger the impact of human capital on employability.

*H5B:* The lower perception reduction of employment opportunities under the COVID-19 epidemic and the stronger the future career clarity of college students, the stronger the impact of social capital on employability.

**Figure 1:**
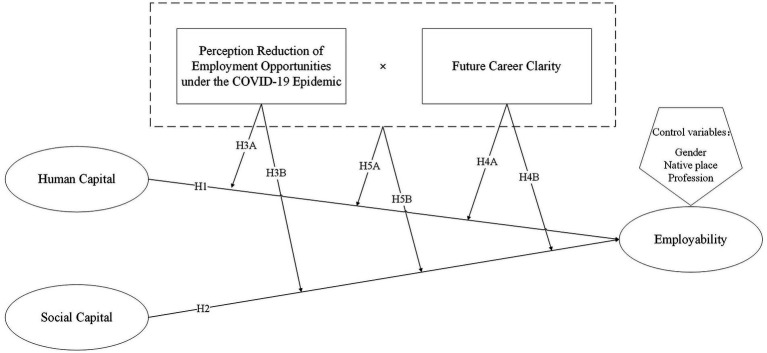
A joint adjustment model of college students’ human capital, social capital, and employability.

To sum up, this study adopts human capital and social capital as independent variables, employability as dependent variables, perception reduction of employment opportunities under the COVID-19 epidemic and future career clarity as moderating variables, and explores (1) the relationship between college students’ human capital, college students’ social capital, and employability; (2) college students’ perception reduction of employment opportunities under the COVID-19 epidemic, future career clarity and their combined effects on college students’ human capital and employability; (3) college students’ perception reduction of employment opportunities under the COVID-19 epidemic, future career clarity and their combined effects on college students’ social capital and employability (Figure 1).

## Materials and methods

### Research sample

Considering the impact of the epidemic, college students who graduated in 2020 and 2021 and have been employed are mainly selected. The survey is mainly divided into three steps. First, the development of the new questionnaire and the reliability and validity test. Because the perception of employment opportunity reduction under the epidemic is a newly developed scale, the main items of the new scale were determined through in-depth interviews and literature methods, and then 89 questionnaires were collected for preliminary reliability and validity tests and preliminary determination of relevant items. The Maturity Scales use the translation back translation program to translate English items into Chinese, and then invites several recent college graduates to read the items and fully communicate with each other, delete or correct the ambiguous parts, collect 198 questionnaires for pre-investigation, and revise the items and structural dimensions of the questionnaire again. Second, by contacting the enrollment and employment offices of major universities, counselors, and class teachers in charge of employment, and using electronic questionnaires, large-scale questionnaires were collected mainly in Sichuan, Chongqing, Shanghai, Jilin, Hunan, and other places. Finally, a large-scale survey was carried out, and 835 questionnaires were finally obtained. According to the completeness of the questionnaire, whether there were contradictions between the positive and negative items, and whether the questionnaire was highly focused on one option, invalid questionnaires were eliminated. A total of 25 invalid questionnaires were eliminated. Finally, 810 valid questionnaires were obtained, and the efficiency of the sample was 97%. In the survey sample, girls accounted for 40.4% and boys accounted for 59.6%; Rural students accounted for 69.6%, and urban registered permanent residence was 30.4%. Literature and history accounted for 4.8%, economy and management 37.7%, science and engineering 37.5%, legal and political 3.3%, foreign languages 2.3%, agriculture and forestry 6.7%, medicine 1.5%, education 1.7% and others 4.4%.

### Measuring tools

#### Measurement of employability

The employability scale developed by Berntson and Marklund (2007) is used, which includes 1 dimension and 5 items. Typical entries include the following three: “My ability is popular in the labor market,” “I can use my social network resources to find a new job that is similar to or better than the current one” and “With my ability, it is relatively easy for me to find a new job that is similar to or even better than the current one in a different organization/company.” Employability was measured on a five-point Likert scale, with a scale of 1 to 5 representing “very unfit” to “strongly fit.” After testing, the scale has good reliability and validity, the Cronbach’s α of the scale is 0.823, the KMO value is 0.844, and the variance explained rate is 58.749%.

### Measurement of human capital

Human capital adopts the measurement method of Qiao et al. (2011) and Wang (2018). The main indicators used in human capital include 10 measurement items: the level of college graduates, the level of college English proficiency certificates, the level of college students’ computer proficiency certificates, the mastery of office software, the ranking of comprehensive evaluation scores, the number of professional-related qualification certificates, the number of professional-related qualification certificates obtained, the highest scholarship level, internship experience, the status of serving as a student cadre, and the level of professional skills are 10 measurement items. The coding is coded from 1 to 5 according to the level from low to high, and then the average level of 10 human capital items is calculated by continuous variable calculation.

### Measurement of social capital

Based on the research of Wang (2016, 2018), social capital is measured by one dimension including five items. Typical items such as: “I know a lot of people who are helpful for my job search,” “most of the people who are helpful for my job search have a good educational background and social status” and “Most of the people who help me with my job search are people I know very well, such as family members or relatives and friends, etc.” Social capital is measured on a five-point Likert scale, with a scale of 1 to 5 indicating “very dissatisfied” to “very good.” After testing, the scale has good reliability and validity, the KMO value is 0.631, the Cronbach’s α of the scale is 0.657, and the cumulative variance explanation rate is 65.429%.

### Measurement of the perception reduction of employment opportunities under the COVID-19 epidemic

College students’ perception reduction of employment opportunities under the COVID-19 epidemic is measured by a self-made questionnaire. There are 6 questions in one dimension. Typical questions are the following six questions: the epidemic has reduced the number of job interviews I have, the epidemic has reduced the quality of my interviews, the epidemic has reduced the number of offers I get, the epidemic has reduced the quality of offers I have gotten, the epidemic has reduced the quality of my offers, and the epidemic has lowered the quality of the vacancies I can find. The perception reduction of employment opportunities under the epidemic is measured on a five-point Likert scale, with a scale of 1 to 5 representing “very unsatisfactory” to “very consistent.” After testing, the scale has good reliability and validity. The Cronbach’s α of the scale is 0.948 (*n* = 810), the KMO value is 0.903 (*n* = 810), and the explained variance ratio is 79.634%.

### Measurement of future career clarity

The future career clarity scale developed by Strauss et al. (2012) was used, with a total of 1 dimension and 5 items. Typical items such as: “I can easily imagine what my future work will be like,” “The future work scene is very clear in my mind” and “I’m very clear about who I want to be in my future work and what I want.” Future career clarity was measured on a five-point Likert scale, with a scale of 1 to 5 representing “very disagree” to “very agree.” After testing, the scale has good reliability and validity, the Cronbach’s α of the scale is 0.924, the KMO value is 0.877, and the variance explained rate is 76.799%.

In this study, background variables such as gender, native place, and major as control variables. Because of occupational gender stereotypes and the physiological characteristics of women’s pregnancy, gender is an important factor affecting employment (Singh et al., 2022), and the employment confidence of males is significantly higher than that of females and there is a significant difference (Ren and Lu, 2021). Therefore, gender is taken as the control variable, in which the female code is 1 and the male code is 2. Because China has obvious differences between urban and rural areas, the difference in educational resources has made the native place an important factor affecting the employability of college students for a long time (Zhong and Wang, 2020). The native place is coded as 1 in rural areas and 2 in urban areas. Major is closely related to the market demand, it is also an important aspect that affects the employment pressure of college students (Yang et al., 2022), major codes were coded as 1–9 according to 9 different categories, and dummy variables were used for data processing.

## Results

### Reliability analysis and common method variances test for the questionnaire

A large sample of about 50% (*n* = 404) of the data was first drawn for exploratory analysis. Because human capital is not measured using latent variables and Likert scales but is calculated using specific relevant indicators, there is no need for reliability and validity tests. The remaining variables were measured using latent variables, Cronbach’s alpha was used to test the reliability of the questionnaire, and the KMO value was used to judge whether it was suitable for factor analysis. The Cronbach’s α of several scales are obtained as: the KMO value of the perception of reduction of employment opportunities under the epidemic is 0.896, the explained variance is 80.796%, and the α value is 0.952; the KMO value of future career clarity is 0.866, the explained variance is 76.562%, and the α value is 0.923; the KMO value of social capital is 0.652, the cumulative variance explained rate is 65.429%, and the α value is 0.675; the employability KMO value is 0.839, the explained variance is 60.770%, and the α value is 0.837. From the above description, we can see that the Cronbach’s α value of all variables is higher than 0.6, the Cronbach’s α values of most scales are higher than 0.8, the KMO value is higher than 0.6, and the KMO values of most scales are higher than 0.8, indicating that the questionnaire has good reliability and is suitable for factor analysis. At the same time, the Harman single factor test method is used to test the homology variance, and all the items in the questionnaire are analyzed by principal component analysis. It is found that the first principal component explains 18.302% of the variance, which is far less than the recommended value of 50%.

### Confirmatory factor analysis

Confirmatory factor analysis was performed on the other half of the samples (*n* = 406) using AMOS22.0, and the specific data are shown in Table 1. From the data in Table 1, it can be seen that the value of χ^2^/*df* in the basic model is much less than 5, the RMSEA value is less than 0.1, the CFI and IFI values are all greater than 0.9, and the NFI, GFI, and AGFI values are all greater than 0.85. The fitting indicators all meet the standard and are significantly better than the four alternative models.

**Table 1:** Confirmatory factor analysis results (*n* = 406).

Model	Description	χ^2/^*df*	CFI	NFI	IFI	GFI	AGFI	RMSEA
Basic model	Five-factor model	1.791	0.948	0.891	0.949	0.899	0.876	0.044
Model 1	Four-factor model	2.233	0.919	0.863	0.919	0.867	0.839	0055
Model 2	Three-factor model	2.884	0.875	0.821	0.876	0.826	0.790	0.068
Model 3	Two-factor model	5.832	0.677	0.637	0.679	0.672	0.608	0.109
Model 4	Single-factor model	7.198	0.585	0.551	0.588	0.584	0.504	0.123

The basic model is the hypothetical model for this study (excluding control variables), including human capital, social capital, the perception reduction of employment opportunities under the COVID-19 epidemic, future career clarity, and employability; Model 1 is human capital + social capital, the perception reduction of employment opportunities under the COVID-19 epidemic, future career clarity, employability; Model 2 is human capital + social capital + the perception reduction of employment opportunities under the COVID-19 epidemic, future career clarity, employability; Model 3 is human capital + social capital + the perception reduction of employment opportunities under the COVID-19 epidemic + future career clarity and employability; Model 4 is human capital + social capital + the perception reduction of employment opportunities under the COVID-19 epidemic + future career clarity + employability.

### Descriptive statistical analysis

Table 2 shows the correlation coefficient, the mean, and the standard deviation of the main research variables. Among them, the first row’s 1–8, respectively, indicate gender, native place, profession, human capital, social capital, employability, perception reduction of employment opportunities under the epidemic, and future career clarity. According to the content of Table 2, it can be seen that gender is closely related to employability, and the employability of female college students is significantly lower than that of male college students (*β* = −0.132, *p* < 0.05); the employability of rural college students is significantly lower than that of urban college students (*β* = 0.072, *p* < 0.1); there is no significant relationship between graduate majors and their employability, but human capital (*β* = 0.111, *p* < 0.05) and social capital (*β* = 0.348, *p* < 0.05) have a significant impact on employability, and both human capital and social capital are significantly positively correlated with college students’ employability. Hypotheses 1 and 2 are preliminarily verified. The perception reduction of employment opportunities under the epidemic is negatively correlated with employability (*β* = −0.101, *p* < 0.05), while future career clarity is significantly positively correlated with employability (*β* = 0.498, *p* < 0.05).

**Table 2:** Means, standard deviations, and correlation analysis results of various variables (*n* = 810).

Variable	1	2	3	4	5	6	7	8
1. Gender	1							
2. Native Place	−0.004	1						
3. Profession	−0.228^**^	−0.035	1					
4. Human Capital	0.247^**^	0.035	−0.157^**^	1				
5. Social Capital	−0.088^*^	0.069^*^	0.016	0.064	(0.657)			
6. Employability	−0.132^**^	0.072^*^	−0.038	0.111**	0.348**	(0.823)		
7. Perception reduction of employment opportunities under the COVID-19 epidemic	0.236^**^	−0.032	−0.179^**^	0.113^**^	−0.036	−0.101^**^	(0.948)	
8. Future career clarity	−0.150^**^	0.065	−0.037	0.042	0.307^**^	0.498^**^	−0.105^**^	(0.924)
Mean	1.60	1.30	4.32	2.5596	3.2886	3.0081	3.2325	3.1721
Standard deviation	0.491	0.460	2.312	0.50315	0.61130	0.61556	0.99289	0.74433

**p < 0.05, *p < 0.1. The data on the diagonal is Cronbach’s Alpha value of the scales.

### Hypothesis testing

The research uses SPSS24.0 and adopts the multi-level regression method. First, test the main effect of human capital and social capital on the employability of college students; secondly, the independent moderating effects of the perception reduction of employment opportunities under the epidemic and future career clarity on the two main effects are tested, respectively. Finally, verify the joint moderating effect of two moderator variables on the relationship between human capital, social capital, and college students’ employability. Before the analysis, all variables are centralized to reduce the possible multicollinearity problem. To better observe the constant term coefficients in the regression analysis, the dependent variable uses uncentralized data. In the table below, human capital is represented by HC, social capital is represented by SC, the variable of perception reduction of employment opportunities under the epidemic is represented by EOR, and future career clarity is represented by FCC.

The Dawson (2014) method was used to test individual and joint moderating effects. When examining the independent moderating effects, formula (1) is used, where y represents the dependent variable (employability), x represents the independent variable (human capital or social capital), and *z* represents the adjustment Variables (perception reduction of employment opportunities or future career clarity).


(1) $$y=b_{0}+b_{1}x+b_{2}z+b_{3}xz+\varepsilon$$

When examining the joint moderating effect, formula (2) is used, where y represents the dependent variable (employability), *x* represents the independent variable (human capital or social capital), and z and w represent Two moderating variables (perception reduction of employment opportunities and future career clarity).


(2) $$y=b_{0}+b_{1}x+b_{2}z+b_{3}w+b_{4}xz+b_{5}xw+b_{6}zw+b_{7}xzw+\varepsilon$$

### The moderating effect of perception reduction of employment opportunities under the COVID-19 epidemic and future career clarity on human capital and employability

As shown in Models 1 and 2 in Table 3, when only the controlled variables are considered, human capital is significantly positively correlated with the employability of college students (*β* = 0.089, *p* < 0.01), and hypothesis 1 is supported. It can be seen from Models 1, 2, 3, and 4 that the product term of human capital and the perception reduction of employment opportunities is significant (*β* = 0.040, *p* < 0.05), so hypothesis 3A is supported. The perception reduction of employment opportunities under the epidemic negatively adjusts the relationship between human capital and employability. The higher the perception reduction of employment opportunities perceived by college students, the weaker the impact of human capital on college students’ employability in the labor market. Models 1, 2, 5, and 6 show that the product term of human capital and future career clarity is significant (*β* = 0.058, *p* < 0.05). Therefore, hypothesis 4A is verified, and college students’ future career clarity positively moderates the relationship between human capital and employability. The stronger the future career clarity of college students, the stronger the impact of human capital on college students’ employability in the labor market. From Models 7, 8, and 9, it is seen that the triple interaction coefficient of human capital, employment opportunity reduction and future career clarity is significant (*β* = −0.025, *p* < 0.05), hypothesis 5A has been verified, and triple interaction adjustment effect exists. That is to say, the lower the perception reduction of employment opportunities under the epidemic perceived by college students, the higher the future career clarity of college students, and the greatest impact of human capital on the employability of college students in the labor market. At this time, college students have the highest employability.

**Table 3:** The moderating effect of perception reduction of employment opportunities under the epidemic and future career clarity on human capital and employability.

Dependent variable: Employability									
Variable	Model 1	Model 2	Model 3	Model 4	Model 5	Model 6	Model 7	Model 8	Model 9
Constant coefficient	3.245	3.288	3.273	3.265	3.159	3.164	3.151	3.149	3.161
Gender	−0.148***	−0.184***	−0.163***	−0.162***	−0.103***	−0.104**	−0.092**	−0.090**	−0.097**
Native place	0.061	0.056	0.052	0.056	0.034	0.022	0.032	0.019	0.018
Profession	−0.018**	−0.014	−0.016*	−0.017*	−0.007	−0.005	−0.009	−0.008	−0.007
HC		0.089***	0.091***	0.092***	0.069***	0.063***	0.071***	0.066***	0.070***
EOR			−0.051**	−0.051**			−0.031	−0.027	−0.028
FCC					0.253***	0.258***	0.251***	0.251***	0.253***
HC* EOR				0.040**				0.026	0.030*
HC*FCC						0.058**		0.057**	0.061**
EOR*FCC								−0.028**	−0.030**
HC*EOR*FCC									−0.025*
*R* ^2^	0.139	0.158	0.164	0.169	0.306	0.316	0.309	0.324	0.327
*R*^2^ changes		0.019	0.006	0.005	0.149	0.010	0.170	0.015	0.003
*F*	32.363***	30.141***	26.294***	23.339***	59.142***	52.943***	51.171***	38.284***	35.236***
*F* changes		18.446***	6.102**	4.853**	172.081***	11.229**	65.824***	5.986***	3.544**
MAX(VIF)	1.063	1.079	1.146	1.147	1.139	1.145	1.169	1.162	1.164

***p < 0.01, **p < 0.05, *p < 0.1. All the coefficients in the table are non-standardized coefficients after data centralization. In the subsequent interaction effect diagram, the dependent variable adopts non-centralized data to better observe the constant term (the same as below). Among them, the change of *R*^2^ and the change of *F*, respectively, represent the change from Model 1 to Model 4, which tests the moderating effect of perception reduction of employment opportunities under the epidemic. The changes from models 1,2 to models 5,6 tested the moderating effect of future career clarity; the changes from Model 7 to Model 9 tested three interactive moderating effects.

**Figure 2:**
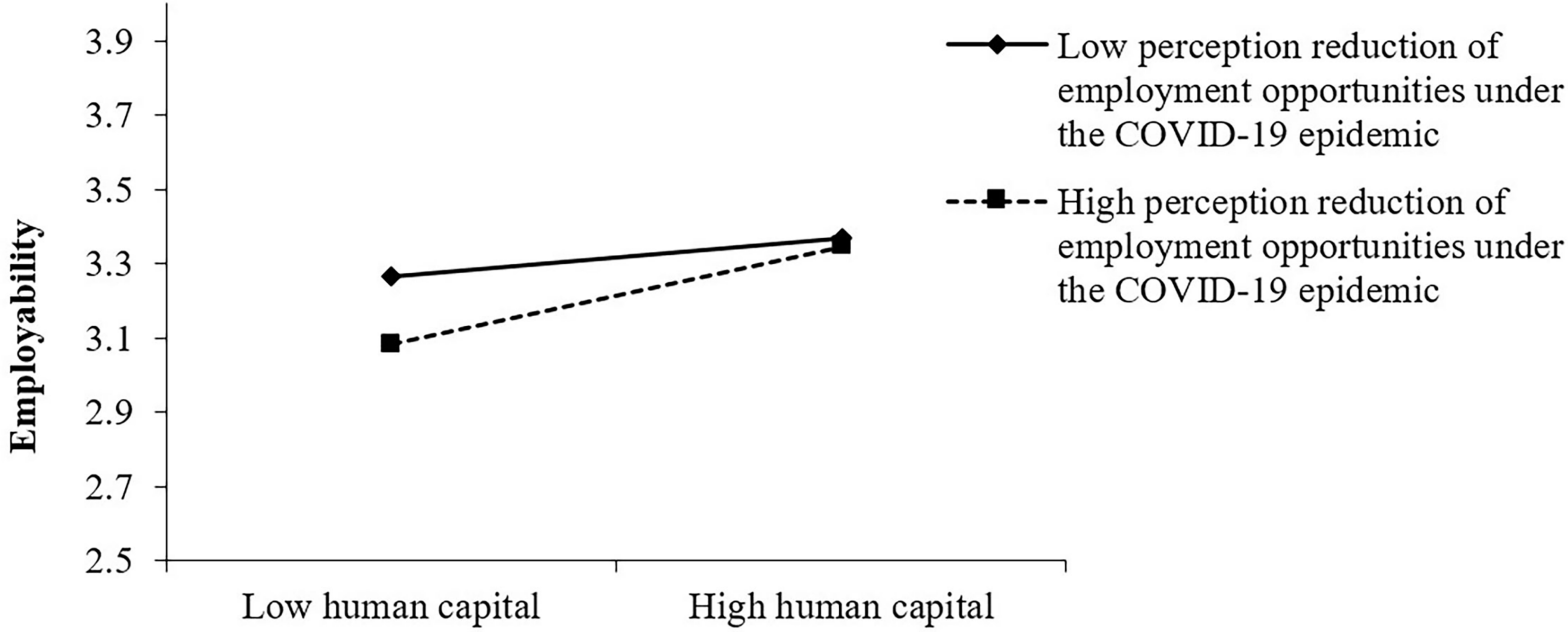
The moderating effect of perception reduction of employment opportunities under the epidemic on human capital and employability.

In this paper, the adjusted variable is divided into groups of high scores and groups of low scores by adding and subtracting one standard deviation from the average of the adjusted variable, and then graphs are drawn. It can be seen from Figure 2 that the perception reduction of employment opportunities under the epidemic negatively moderates the impact of human capital on college students’ employability. When college students perceive a higher perception reduction of employment opportunities under the epidemic, the impact of human capital on employability is smaller.

**Figure 3:**
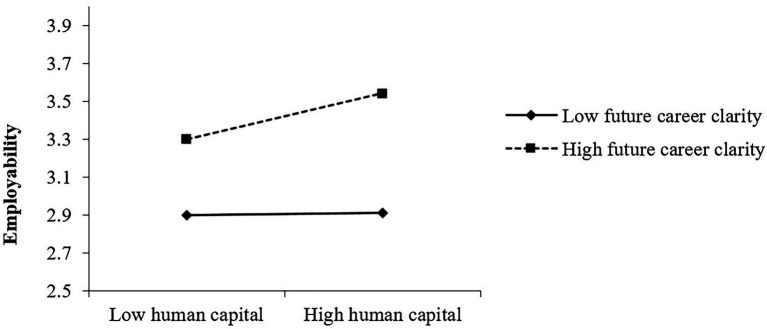
The moderating effect of future career clarity on human capital and employability.

It can be seen from Figure 3 that college students’ future career clarity positively moderates the relationship between human capital and their employability. The stronger the future career clarity of college students, the higher the impact of human capital on the employability of college students in the labor market.

**Figure 4:**
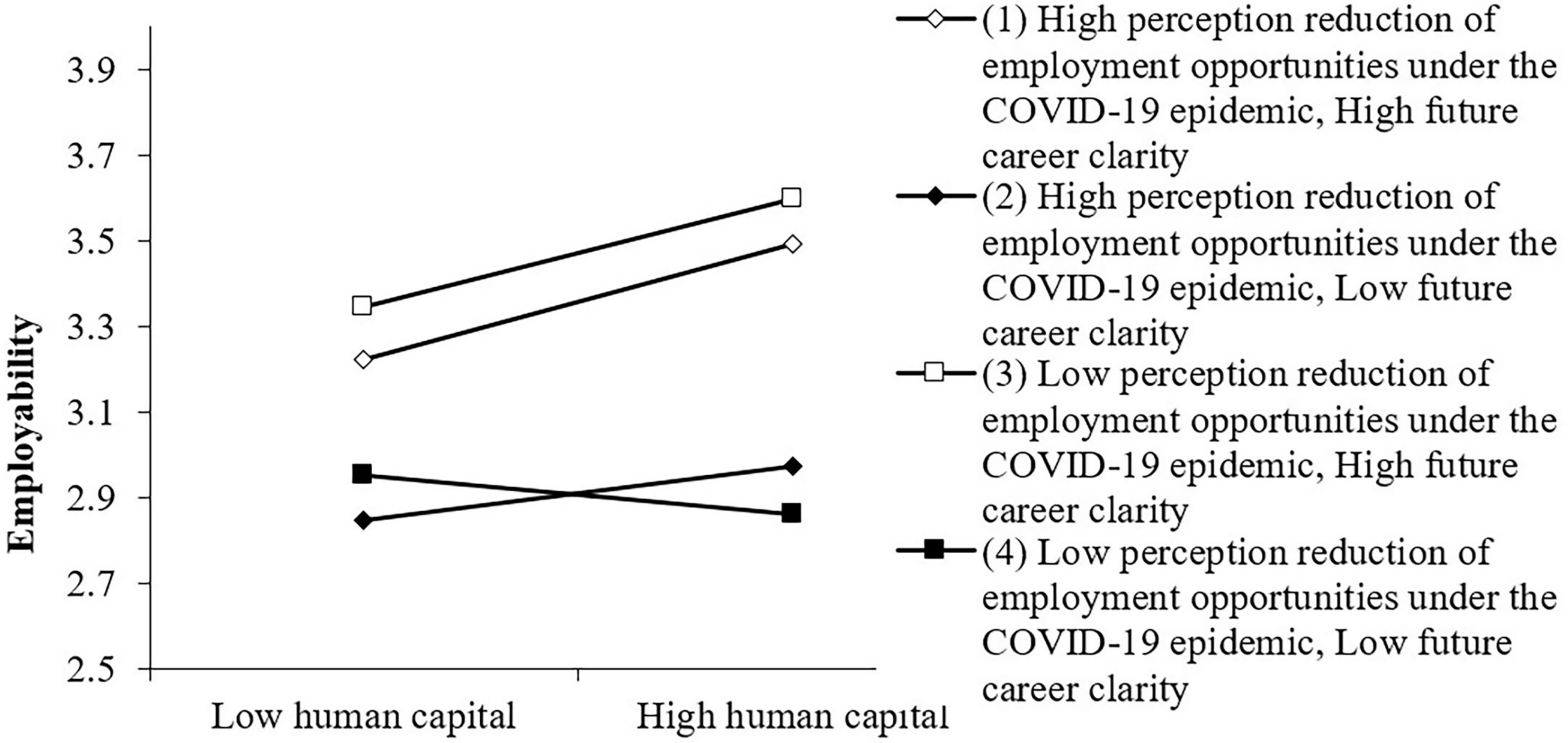
The joint moderating effect of perception reduction of employment Opportunities under the COVID-19 epidemic and future career clarity on human capital and Employability.

It can be seen from Table 3 and Figure 4 that there is a triple interactive moderating effect, that is, the lower the perception reduction of employment opportunities under the epidemic perceived by college students, and the higher the future career clarity of college students, the human capital has the greatest impact on college students’ employability. At this time, the employability of college students is the highest, and this data is completely consistent with the hypothesis.

### The moderating effect of perception reduction of employment opportunities under the COVID-19 epidemic and future career clarity on social capital and employability

As shown in Models 10 and 11 in Table 4, when only the controlled variables are considered, social capital is significantly positively correlated with the employability of college students (*β* = 0.207, *p* < 0.01), and H2 is supported. From models 10, 11, 12, and 13, it can be seen that the product term of social capital and reduction in employment opportunities is not significant (*β* = 0.016; *p* > 0.1), so H3B is not supported. It can be seen from Models 10, 11, 14, and 15 that the product term of social capital and future career clarity is not significant (*β* = −0.002, *p* > 0.1), so Hypothesis 4B has not been verified. It can be seen from Models 16, 17, and 18 that the triple interaction coefficient between social capital and employment opportunities reduction under the epidemic situation and future career clarity is significant (*β* = 0.014, *p* < 0.05). Hypothesis 5B has been verified, and the triple interaction adjustment effect exists. That is to say, the lower the perception reduction of employment opportunities under the epidemic perceived by college students, the higher the future career clarity of college students, and the social capital has the greatest impact on college students’ employability in the labor market. At this time, college students’ employability is the highest.

**Table 4:** The moderating effect of perception reduction of employment opportunities under the COVID-19 epidemic and future career clarity on social capital and employability.

Dependent variable: Employability									
Variable	Model 10	Model 11	Model 12	Model 13	Model 14	Model 15	Model 16	Model 17	Model 18
Constant coefficient	3.265	3.245	3.230	3.226	3.122	3.123	3.115	3.109	3.093
Gender	−0.185***	−0.148***	−0.128**	−0.127***	−0.074*	−0.074*	−0.063	−0.058	−0.053
Native place	0.092**	0.061	0.057	0.058	0.037	0.037	0.035	0.033	0.036
Profession	−0.019*	−0.018**	−0.021**	−0.021**	−0.010	−0.010	−0.012	−0.012	−0.012
SC		0.207***	0.206***	0.208***	0.131***	0.131***	0.131***	0.135***	0.135***
EOR			−0.048**	−0.047**			−0.028	−0.024	−0.032
FCC					0.259***	0.259***	0.257***	0.252***	0.253***
SC* EOR				0.016				0.022	0.025
SC*FCC						−0.002		−0.003	0.004
EOR*FCC								−0.031**	−0.028*
SH* EOR*FCC									0.014*
*R* ^2^	0.027	0.139	0.144	0.145	0.290	0.290	0.297	0.301	0.304
*R*^2^ changes		0.111	0.006	0.001	0.156	0.000	0.270	0.005	0.003
*F*	7.493	32.363	27.076	22.754	67.225	55.957	56.450	38.330	34.871
*F* changes		104.097	5.246	1.231	178.179	0.026	102.574	1.766	2.915
MAX(VIF)	1.056	1.063	1.108	1.110	1.110	1.134	1.139	1.152	1.155

***p < 0.01, **p < 0.05, *p < 0.1.Among them, the change of *R*^2^ and the change of *F*, respectively, represent the change from Model 10 to Model 13, which tests the moderating effect of perception reduction of employment opportunities under the epidemic. The changes from models 10,11 to models 14,15 tested the moderating effect of future career clarity; the changes from Model 16 to Model 18 tested three interactive moderating effects.

**Figure 5:**
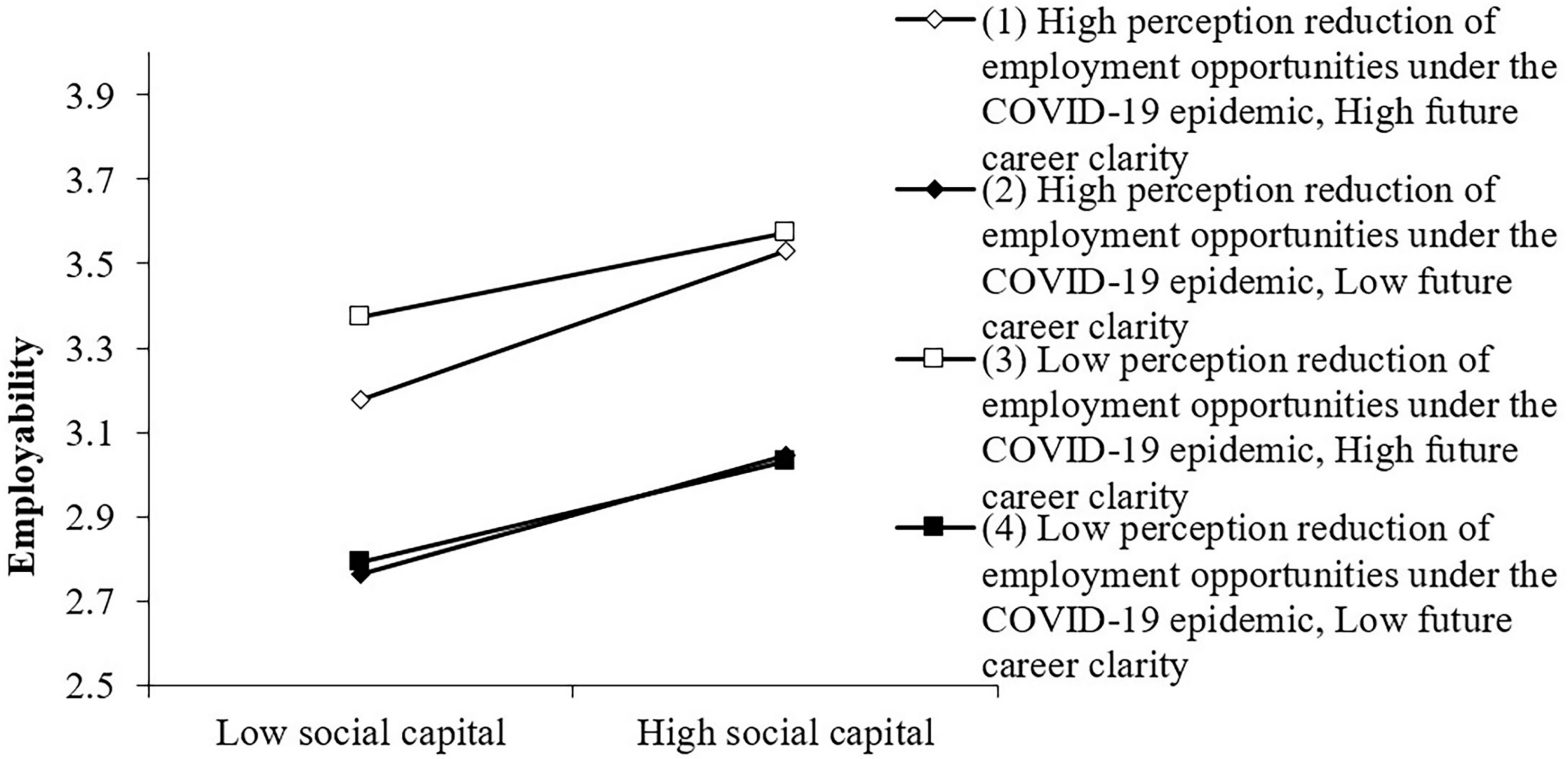
The joint adjustment effect of perception reduction of employment Opportunities under the COVID-19 epidemic and future career clarity on social capital and employability.

It can be seen from Table 4 and Figure 5 that triple interaction regulation exists. That is to say, the lower the perception reduction of employment opportunities under the epidemic perceived by college students, the higher the future career clarity of college students, and the social capital has the greatest impact on college students’ employability in the labor market. At this time, college students’ employability is the highest.

## Discussion

### Research conclusion

Based on the background of the epidemic, this study constructs a theoretical model of the relationship between human capital and social capital and college students’ employability and conducts empirical analysis, and draws the following conclusions: (1) Both human capital and social capital have a positive and significant impact on the employability of college students, and the impact of social capital on the employability of college students is greater than the impact of human capital on the employability of college students. (2) The perception of reduction of employment opportunities under the COVID-19 epidemic negatively regulates the relationship between human capital and college students’ employability. That is to say, in the context of the epidemic, the higher the perception reduction of employment opportunities perceived by college students, the smaller the positive impact of human capital on college students’ employability. Moreover, the perception reduction of employment opportunities under the COVID-19 epidemic has no moderating effect between social capital and employability. (3) The future career clarity positively regulates the relationship between human capital and college students’ employability, that is the higher the future career clarity of college students, the greater the positive impact of human capital on college students’ employability in the labor market. (4) the perception reduction of employment opportunities under the COVID-19 epidemic and the future career clarity jointly adjust the relationship between human capital, social capital, and college students’ employability. When the perception reduction of employment opportunities under the COVID-19 epidemic is lower and the future career clarity is higher, the positive impact of human capital and social capital on the employability of college students is greater.

### Theoretical significance

First, it expands domestic theoretical and empirical research on college students’ employability. The previous research on the employability of college students mainly focused on the general discussion of the concept, structure, and training strategies of employability (Gong and Cai, 2018; Mao and Li, 2021), and lacked empirical analysis research. Existing empirical research also focuses on demonstrating the correlation and influence degree between college students’ employability and college students’ employment (Yang, 2017), as well as the influencing factors of college students’ employment ability improvement (Cheng, 2017). Due to the different national conditions between China and the West, the localization of “employability” in college students needs to be further explored, and in the context of the epidemic, how to cultivate and improve the employability of college students is undoubtedly one of the most important issues to be solved. This study constructs a model of the relationship between human capital, social capital, and college students’ employability, and introduces two moderating variables: perception reduction of employment opportunities under the COVID-19 epidemic and future career clarity. This study explores the impact of human capital and social capital on college students’ employability under the epidemic, deepens the practical application value and practical guiding significance of college students’ employability, and tests and promotes related theoretical research on college students’ employability.

Secondly, it enriches the research on the employment of college students from the dual perspectives of social capital and human capital. Since Xu (2002) first brought the three themes of “human capital,” “social capital” and “college student employment” into the same research system, many scholars have explored the relationship between human capital, social capital, human capital, and social capital on the employment of college students. Research perspectives and themes are gradually showing a trend of diversification, and research is gradually deepening, from employment rate to employment quality, from a single dimension to specific indicators (Hu and Fei, 2019; Wang, 2020). The employability of college students is one of the internal reasons affecting the employment of college students, but little attention has been paid to the impact of human capital and social capital on the employability of college students. This paper studies the influence mechanism on employability from the perspectives of “personal ability” and “social capital,” and compares the difference in influence, which not only provides empirical support for “which is more important,” but also reflects from the side that the employability of college students is a developmental concept, that is, the employability of college students are affected by the joint action of relationship and ability, and has the characteristics of dynamic and stage. This study also responds to Xin’s (2019) expectation of expanding employment-related indicators, filling the gap in the research on the impact of human capital and social capital on college students’ employability.

Finally, this paper explores the moderating effects of perception reduction of employment opportunities under the COVID-19 epidemic and future career clarity on college students’ social capital, human capital, and employability. Although the terms “perception reduction of employment opportunities under the COVID-19 epidemic” and “perception reduction of employment opportunities “have not directly appeared in previous research, there is a concept of “perception of external job opportunities” from a subjective perspective (Tang et al., 2019). Under the epidemic environment, the ability of enterprises to absorb employment has seriously declined, and college students’ perception reduction of employment opportunities is beyond doubt. In addition, scholars in the past have mainly focused on examining the influencing factors and effects of job search clarity, such as proactive personality as the influencing factor (Wang and Cheng, 2022), employability as the effect result (Ye et al., 2017), but they are less regarded as situational moderating factors. This study empirically examines the moderating effects of two variables, perception reduction of employment opportunities under the COVID-19 epidemic and future career clarity, between college students’ social capital, human capital, and employability. This study comprehensively examines the mechanism of social capital and human capital on employability from the perspective of internal and external perception, which can provide a reference for the government, schools, and colleges to effectively improve the employability of college students.

### Practical significance

First, college students should strive to improve their human capital and social capital to enhance their employability. Research shows that both human capital and social capital have a significant impact on college students’ employability. The improvement of employability is the result of long-term accumulation. For college students, if they want to improve their employability, they need to focus on learning their professional knowledge, the improvement of professional skills, and accumulating human capital such as participation in internship practice activities during school, to become suitable candidates for employers. In addition, Fei Xiaotong believes that the relationship between people in traditional Chinese society is a pattern of differential order, and the way of doing things depends on the alienation of “relationship.” Therefore, in the context of China, the important role of social capital is more prominent. College students should take the initiative to contact society while they are in school, expand their social network relationships, obtain more social resources, and pay more attention to the establishment and maintenance of their social relationships. Thereby enhancing future employability in the labor market and improving the quality of employment.

Second, the government should increase policy support to help college students find jobs during the epidemic. College students’ perception of the external environment is an important contingency factor to change their employability. Therefore, increasing employment assistance for college graduates can help create a social environment that promotes employment and weaken the perception of the reduction of employment opportunities. In the post-epidemic period, governments at all levels should always take helping college graduates solve the problem of stable employment as an important matter. Based on implementing and improving the active employment promotion policies that have been introduced, the government should introduce a new round of policies and measures to promote the employment of college graduates, strengthen the publicity and interpretation of the policies, promote the implementation of the policies to be effective, and give better play to the effects of policy stabilization and employment expansion. In addition, SMEs are the main channel for absorbing employment and the main body of “stabilizing employment.” The government needs to optimize the incentive measures for small and medium-sized enterprises, strive to create a large number of jobs suitable for college students, expand the employment scale of college students, and give full play to the role of enterprise talent “reservoir.”

Third, colleges and universities should guide students to correctly recognize themselves and improve their employability. College students’ understanding of themselves is also an important contingency factor for the improvement of college students’ employability. Therefore, according to social needs and conditions, college students need to make their career plans as soon as possible for smooth employment in the future. The relevant education departments of schools should provide career guidance to them at the university stage to improve their career adaptability and help them determine their career goals. College students with clear employment goals can prepare for employment during the study period, learn and master the knowledge and skills required for the target occupation in time, and pay attention to the relevant employment information and channels of the target occupation at any time. And college students can compare their current behavioral ability with the behavioral ability required by the target occupation, so that college students are more motivated to produce corresponding employment behaviors, and thus more easily obtain employability.

### Research limitations and prospects

First, the reliability and validity of the newly developed scale need to be further tested. Since few scholars have studied the employment of college students under the epidemic, there is no mature scale for the new concept of “perception reduction of employment opportunities under the COVID-19 epidemic.” Therefore, this study refers to relevant literature and compiles a scale of the perceived reduction of employment opportunities under the COVID-19 epidemic. The reliability and validity of the scale in this study are acceptable, but further tests on the reliability and validity of the questionnaire are needed in the later stage.

Second, there are certain limitations in research samples and data measurement methods. A total of 810 valid questionnaires were obtained through team resources. In the future, larger-scale data can be obtained through third-party research institutions, or scientific sampling methods can be used to expand the sample size to avoid bias in research results. In addition, the research data were obtained through the self-assessment method of college students, and the relationship between variables may be affected by homologous variance, but the results of Harman single factor analysis and confirmatory factor analysis confirmed that there is no serious common method bias. In future research, data can be collected from more different ways and sources depending on the nature of the variables themselves. For example, data of different variables can be collected in stages at different time nodes. For example, multi-party evaluation data from families and schools can be used for future career clarity.

Finally, the study delved into the boundary mechanism but did not explore the mediation mechanism. The study discusses the contingency effects of two important variables, the perception reduction of employment opportunities under the COVID-19 epidemic and future career clarity, but does not deeply explore the impact of human capital and social capital on college students’ employability. The “black box” of the relationship between these three needs to be further explored.

## Data availability statement

The raw data supporting the conclusions of this article will be made available by the authors, without undue reservation.

## Author contributions

YS: overall conception and writing of the thesis. YJ: thesis idea. XJ: language expression and graphic production. ZY: thesis writing. YL: essay writing and overall conception. LH: theoretical exposition. LW, CH, HG, and CJ: data collection. All authors contributed to the article and approved the submitted version.

## Funding

This research was supported by Humanities and Social Sciences Research of the Ministry of Education (22XJC630008); China Academic Degree and Graduate Education Society (2021-NLZX-YB94); Sichuan Provincial Department of Education (CJSFZ21-09); Sichuan University Students’ Ideological and Political Research Center (CSZ21004); Sichuan Agricultural University Social Science Association (2020PTZD02); Sichuan County Economic Development Research Center (XY2021022); Sichuan Revolutionary Old Area Development Research Center (SLQ2021SB-05).

## Conflict of interest

The authors declare that the research was conducted in the absence of any commercial or financial relationships that could be construed as a potential conflict of interest.

## Publisher’s note

All claims expressed in this article are solely those of the authors and do not necessarily represent those of their affiliated organizations, or those of the publisher, the editors and the reviewers. Any product that may be evaluated in this article, or claim that may be made by its manufacturer, is not guaranteed or endorsed by the publisher.
